# Assessing the burden of intestinal parasites affecting newly arrived immigrants in Qatar

**DOI:** 10.1186/s13071-016-1906-6

**Published:** 2016-12-01

**Authors:** Marawan A. Abu-Madi, Jerzy M. Behnke, Ahmed Ismail, Sonia Boughattas

**Affiliations:** 1 Department of Biomedical Science, College of Health Sciences, Biomedical Research Center, Qatar University, P.O. Box 2713, Doha, Qatar; 2 School of Life Sciences, University of Nottingham, University Park, Nottingham, NG7 2RD UK; 3 Medical Commission, Ministry of Public Health, P.O. Box 42, Doha, Qatar

**Keywords:** New immigrants, Helminth, Protozoa, *Blastocystis*, *Giardia*, Qatar

## Abstract

**Background:**

In the last decades, the enormous influx of immigrants to industrialized countries has led to outbreaks of parasitic diseases, with enteric infections being amongst the most frequently encountered. In its strategy to control such infection, Qatar has established the Pre-Employment Certificate (PEC) program which requires medical inspection before arrival in Qatar and which is mandatory for immigrant workers travelling to the country. To assess the reliability of the PEC, we conducted a survey of intestinal parasites, based on examination of stool samples provided by immigrant workers (*n* = 2,486) recently arrived in Qatar.

**Results:**

Overall prevalence of helminths was 7.0% and that of protozoa was 11.7%. Prevalence of combined helminths was highest among the western Asians and the highest prevalence of combined protozoan parasites was among workers from North to Saharan Africa. Analysis of temporal changes showed an increasing trend of protozoan infections over the investigated 3 years. A major contribution to this temporal change in prevalence came from *Blastocystis hominis* as well as from other protozoan species: *Giardia duodenalis* and *Endolimax nana*. Analysis of the temporal trend in species richness of the protozoan species showed a significant increase in the mean number of species harboured per subject across this period.

**Conclusion:**

The increase of protozoan infections over recent years raises some concerns. It suggests that screening protocols for applicants for visas/work permits needs to be revised giving more careful attention to the intestinal protozoan infections that potential immigrants may harbor.

## Background

The burden of intestinal parasitic disease remains one of the greatest health problems in the developing world. The World Health Organization estimates that about 3.5 billion people worldwide are still affected by intestinal parasitic infections [1]. Reports have already described the role of immigrants in outbreaks of parasitic diseases in industrialized countries [2]. In recent years, the changing profile of immigration into industrialized countries has seen an increasing number of people arriving from developing countries with the number of arrivals increasing by even more than 50% in some countries [3]. Migrants from developing countries are mostly young healthy individuals, looking for work opportunities. However, among them and among refugees escaping war-torn zones, significant numbers have been reported to carry infectious diseases [2]. Infection is largely determined by the geographical origin, the ethnicity and by the environmental and social conditions at the departure point of migrants and during the migratory route [4]. Intestinal parasitosis is one of several immigrants’ health concerns identified by the US Center for Disease Control [5]. In fact, given appropriate conditions for transmission, importation of parasites by mobile populations into new geographical areas can even lead to outbreaks of disease in the host country and reintroduction of infections that had been eradicated previously from those countries [6]. Published infection rates for intestinal parasites among immigrant populations have been reported to range from 29% to as high as 81% in some cases [7].

Qatar has drawn vast numbers of immigrant workers in the last two decades, often from countries with poor socioeconomic levels, looking for better opportunities [8]. With this mass influx of immigrants, patent intestinal parasitic infections have been observed and monitored. Worryingly, high prevalence of both helminth and protozoan infections was reported in the period 2005 to 2008, among settled immigrants and long-term residents in Qatar [9], which led to changes in the laws governing health inspection and associated treatment of immigrants. The pre-employment certificate (PEC) was established in 2009 and is provided at local Qatar embassy-approved clinical centers in the countries of origin of immigrants and hence before arrival in Qatar. It requires medical checks that include stool examination and any applicant with persistent infection will be denied an entry visa to Qatar.

A subsequent report spanning the decade to 2014 for both helminths [10] and protozoan [11] infections, showed that settled immigrants from Asia, especially those from West Asia/Nepal, were associated with the highest prevalence of intestinal helminth infections (notably hookworms) and also specific intestinal protozoa (e.g. *Giardia duodenalis*) [9, 12], even after acquiring residency status in Qatar. Therefore, between 2012 and 2014 we carried out a survey of recent arrivals, conducting stool examinations to identify the key risk factors that were responsible for the high prevalence of gastro-intestinal parasites among this regional group of immigrants. Here, we report on the prevalence and abundance of intestinal helminth and protozoan infections among immigrant workers newly arrived to Qatar.

## Methods

The present study was based on a cross-sectional survey of intestinal parasitic infections among recently arrived immigrants (*n* = 2,486) during the period 2012–2014. Random stool samples were collected during routine health examinations of individuals who, soon after arrival in the country, reported to the Medical Commission in order to obtain work permits. Personal data were carefully recorded for each subject, and sample collection and stool examination were processed as previously described [8]. Briefly, stool examination was carried out in a safety cabinet in the Laboratory of the Medical Commission, where 5 g of stools from each sample were preserved in an Ecofix preservative vial (Meridin Biosciences Inc., Cincinnati, USA). The contents were stirred with fine clean disposable wooden sticks to break up large clumps and mixed vigorously by vortex to homogenize the sample. To ensure adequate fixation of the homogenized stool, the sample was kept for half an hour at room temperature. The preserved sample was mixed by vortex and filtered through a macro-con filtration unit for the removal of bulky debris that could not be broken down further. After filtration, 10% formalin and ethyl acetate were added, the sample was centrifuged for 10 min at 3,000 rpm and the fluid containing diethyl ether and formalin was discarded. The pellet was re-suspended in 1 ml by agitation and triplicate 10 μl aliquots of the re-suspension were poured onto a microscope slide containing one drop of iodine. These were examined microscopically for the presence/absence of parasite eggs/cysts to enable identification and quantification of parasites in positive samples. Care was taken at this stage to distinguish worm eggs/ova and protozoan cysts from other faecal detritus in the specimen. Parasites were identified by egg/cyst morphology and recorded as present/absent for each species as reported elsewhere [13]. All eggs/cysts that were observed in each sample were measured, photographed and each type was counted separately. All stool samples were examined by experienced professional technicians.

For quantitative data, reflecting abundance of infection, three aliquots of 10 μl were examined from each tube. Egg and cyst counts were expressed as eggs per gram of faeces (EPG) or cysts/oocysts per gram of faeces (CPG). The average number of these counts was calculated and the number of eggs and cysts/oocysts per gram was calculated using the following equation:

Egg/cyst or larvae per gram = 1,000*X/10*5 where X is the average number of parasites; 5 is the mass of stool specimen; 1,000 is the total volume of the sediment (1 ml); and 10 is the volume of the sample examined (10 μl).

The means of EPG and CPG (± standard error of the mean = SEM) were calculated from all samples within specific data subsets (including also all subjects without eggs/cysts as recommended [14, 15]).

### Statistical analysis

Prevalence data are shown with 95% confidence limits (CL_95_), calculated as described by Rohlf & Sokal [16] employing bespoke software. Prevalence was analysed by maximum likelihood techniques based on log-linear analysis of contingency tables using the software package IBM SPSS Statistics (Version 21). Initially, full factorial models were fitted, incorporating as factors sex (SEX at 2 levels, males and females), age (AGE CLASS at 4 levels: Age class 1 [16–22 years, *n* = 303]; Age class 2 [23–29 years, *n* = 856]; Age class 3 [30–37 years, *n* = 823] and age class 4 [38–58 years, *n* = 504]) and region of origin (REGION at 4 levels, as defined below). The study population (*n* = 2,486) came from 24 countries which we allocated to four geographical regions for ease of initial analysis. These were eastern Asia (*n* = 936) comprising Indonesia (*n* = 528), Myanmar (*n* = 1), Philippines (*n* = 396), Thailand (*n* = 9) and Vietnam (*n* = 2); western Asia (*n* = 1,289) comprising Bangladesh (*n* = 192), India (*n* = 433), Nepal (*n* = 373), Pakistan (*n* = 5) and Sri Lanka (*n* = 286); northern and Saharan Africa (*n* = 138) comprising Chad (*n* = 1), Egypt (*n* = 9), Eritrea (*n* = 11), Ethiopia (*n* = 107), Morocco (*n* = 5) and Sudan (*n* = 5); sub-Saharan Africa (*n* = 123) comprising Burkina Fiasco (*n* = 1), Cameroon (*n* = 3), Ghana (*n* = 14), Kenya (*n* = 73), Nigeria (*n* = 26), Tanzania (*n* = 1), Uganda (*n* = 4) and Zimbabwe (*n* = 1).

For each species of parasite, and for higher taxa, the presence/absence of parasites (INFECTION) was coded as a binary factor. These explanatory factors were fitted initially to all models that were evaluated. For each level of analysis in turn, beginning with the most complex model, involving all possible main effects and interactions, those combinations that did not contribute significantly to explaining variation in the data were eliminated in a stepwise fashion beginning with the highest-level interaction (the backward selection procedure). A minimum sufficient model was then obtained, for which the likelihood ratio of χ^2^ was not significant, indicating that the model was sufficient in explaining the data. The importance of each term (i.e. interactions involving infection) in the final model was assessed by the probability that its exclusion would alter the model significantly and these values relating to interactions that included presence/absence of infection (INFECTION) are given in the text [13]. Where relevant we also fitted in turn models with just one of the factors and INFECTION.

Species richness (number of species harboured by each subject) was analysed by generalized linear models (GLM) based on Poisson errors and we report the value of Wald χ^2^.

Abundance of infection was analysed only by non-parametric tests (Mann–Whitney *U* test for two samples, for each of which we report the value of *z*, and Kruskal-Wallis test for more than 2 samples, for each of which we report the value of *H*), since with so many uninfected subjects in the study group, statistical analysis based on general linear models (raw or suitably transformed data assuming Gaussian distribution of errors) or generalized linear models (negative binomial errors) was not satisfactory. Assessment of the goodness of model fits was based on the distribution of residuals, Q-Q plots and *R* ^2^ values.

## Results

### Demography of the study group

The data in Table 1 summarize the key demographic information of the study group and show that there were almost 9.0% more females than males in the study group, and that the youngest age class was under-represented compared to the three older age classes. Moreover, the distributions of host sex (REGION × SEX, χ^2^_3_ = 908.1, *P* < 0.001) and age (REGION × AGE CLASS, χ^2^_9_ = 214.5, *P* < 0.001) across the four geographical regions were not uniform, and this uneven distribution of subjects is taken into account in further analyses.

**Table 1:** Distribution of the study group by region of origin, sex and age class

	western Asia (*n* = 1289)	eastern Asia (*n* = 936)	northern & Saharan Africa (*n* = 138)	sub-Saharan Africa (*n* = 123)	Total (%)
Sex					
Males	921	118	18	78	1135 (45.7)
Females	368	818	120	45	1351 (54.3)
Age class (age range)					
1 (16–22)	216	33	38	16	303 (12.2)
2 (23–29)	414	311	67	64	856 (34.4)
3 (30–37)	375	391	21	36	823 (33.1)
4 (38–58)	284	201	12	7	504 (20.3)

### Prevalence of infection

#### Prevalence of parasites by region of origin of subjects

Table 2 shows the prevalence of infections with each of the seven helminth taxa, the eight protozoan taxa, combined helminths, combined protozoa and all parasites combined. Prevalence of combined helminths was highest among the western Asians but also very close among those from eastern Asian countries although the difference between regions was not significant (χ^2^_3_ = 1.47, *P* = 0.690). For western Asian countries, hookworms accounted for most of the infections and there was a significant difference in prevalence of hookworms between the four regions (REGION × INFECTION, χ^2^_3_ = 10.5, *P* = 0.015) whereas for eastern Asians it was a combination of hookworms, *Ascaris lumbricoides* and *Trichuris trichiura*. However, the prevalence of *A. lumbricoides* did not vary significantly between the regions (REGION × INFECTION, χ^2^_3_ = 2.86, *P* = 0.413) nor did that of *T. trichiura* (REGION × INFECTION, χ^2^_3_ = 2.23, *P* = 0.526).

**Table 2:** Prevalence % (CL_95_) of helminth and protozoan parasites by region of origin of the subjects in the study

	western Asia	eastern Asia	northern & Saharan Africa	sub-Saharan Africa	Overall
Helminths					
*Ascaris lumbricoides*	1.4 (0.83–2.21)	**2.4** (1.35–4.01)	1.4 (0.33–5.03)	1.6 (0.44–5.03)	1.8 (1.29–2.38)
Hookworms	**4.7** (3.62–6.08)	2.4 (1.35–4.01)	2.2 (0.66–6.16)	1.6 (0.44–5.03)	3.5 (2.84–4.36)
*Trichuris trichiura*	1.2 (0.71–2.02)	**1.8** (0.97–3.35)	0.7 (0.10–3.81)	0.8 (0.12–3.71)	1.4 (0.98–1.96)
*Strongyloides stercoralis*	**0.5** (0.17–1.01)	0.5 (0.22–1.55)	0.0 (0.0–2.58)	0.0 (0.0–2.30)	0.4 (0.22–0.79)
*Taenia* spp.	0.1 (0.0–0.43)	0.0 (0.0–0.70)	**0.7** (0.10–3.81)	0.0 (0.0–2.30)	0.1 (0.01–0.29)
*Hymenolepis nana*	0.5 (0.17–1.01)	0.2 (0.09–1.04)	0.7 (0.10–3.81)	**0.8** (0.12–3.71)	0.4 (0.19–0.74)
*Enterobius vernicularis*	**0.2** (0.02–0.56)	0.1 (0.04–0.87)	0.0 (0.0–2.58)	0.0 (0.0–2.30)	0.1 (0.02–0.35)
All helminths combined	**7.3** (5.89–8.92)	7.2 (5.21–9.66)	5.8 (2.98–10.78)	4.9 (2.43–9.33)	7.0 (6.03–8.05)
Protozoa					
*Blastocystis hominis*	5.7 (4.44–7.12)	4.7 (3.19–6.82)	**10.9** (6.59–17.03)	4.1 (1.81–8.26)	5.5 (4.61–6.41)
*Chilomastix mesnili*	0.1 (0.0–0.43)	0.0 (0.0–0.70)	**1.4** (0.33–5.03)	0.0 (0.0–2.30)	0.1 (0.02–0.35)
*Endolimax nana*	3.2 (2.28–4.32)	2.1 (1.20–3.75)	4.3 (1.94–8.97)	**8.9** (5.39–14.42)	3.1 (2.48–3.92)
*Entamoeba coli*	2.2 (1.51–3.23)	1.3 (0.61–2.65)	**8.7** (5.03–14.50)	1.6 (0.44–5.03)	2.2 (1.67–2.88)
*Entamoeba hartmanni*	0.5 (0.22–1.12)	0.3 (0.13–1.21)	**0.7** (0.10–3.81)	0.0 (0.0–2.30)	0.4 (0.22–0.79)
*Entamoeba histolytica*/*dispar*	1.1 (0.59–1.82)	0.6 (0.27–1.72)	**1.4** (0.33–5.03)	0.8 (0.12–3.71)	0.9 (0.59–1.39)
*Giardia duodenalis*	**3.6** (2.61–4.76)	1.1 (0.46–2.37)	1.4 (0.33–5.03)	0.0 (0 − 2.30)	2.3 (1.77–3.02)
*Iodamoeba butschlii*	0.2 (0.05–0.68)	**0.3** (0.13–1.21)	0.0 (0.0–2.58)	0.8 (0.12–3.71)	0.3 (0.11–0.58)
All protozoa combined	12.7 (10.90–14.54)	8.5 (6.44–11.25)	**21.7** (15.75–29.12)	13.0 (8.56–19.02)	11.7 (10.4–12.93)
Helminths and protozoa combined	19.2 (17.01–21.31)	14.6 (11.85–17.88)	**26.8** (20.15–34.54)	17.1 (11.90–23.70)	17.8 (16.28–19.28)

The highest regional prevalence for each species is in bold

The highest prevalence of combined protozoan parasites was among subjects from northern and Saharan Africa, and this was almost twice as high as values from any of the other three regions (Table 2; for REGION × INFECTION, χ^2^_3_ = 22.4, *P* < 0.001). Subjects from northern and Saharan Africa had the highest prevalence of six of the eight species of protozoa recorded in our study, with a particularly high prevalence of *Entamoeba coli*.

*Blastocystis hominis* showed only a weakly significant difference in prevalence between the regions when fitted alone with REGION (REGION × INFECTION, χ^2^_3_ = 7.85, *P* = 0.049) but was also a component of an interaction with SEX in the minimum sufficient model after initial fitting of all the factors (REGION × SEX × INFECTION, χ^2^_3_ = 8.17, *P* = 0.043). This interaction arose because in two regions prevalence was slightly lower in female subjects and in two higher. The biggest discrepancy between the sexes was among subjects from eastern Asia, where prevalence among males was low at 0.8% (CL_95_ = 0.13–3.68) but higher among female subjects at 5.3% (CL_95_ = 3.74–7.30).

The prevalence of *Endolimax nana* varied significantly between regions (REGION × INFECTION, χ^2^_3_ = 13.3, *P* = 0.004), and as Table 2 shows prevalence was particularly high among subjects from sub-Saharan Africa. That of *G. duodenalis* also varied significantly between regions (REGION × INFECTION, χ^2^_3_ = 8.94, *P* = 0.030), but the highest prevalence was among subjects from western Asia.

The prevalence of *E. coli* varied between regions in a model with just REGION × INFECTION (χ^2^_3_ = 20.1, *P* < 0.001), as shown in Table 2, but when other factors were fitted, the only significant term was SEX × REGION × INFECTION (χ^2^_3_ = 8.063, *P* = 0.045). This arose because the only infected male subjects were from western Asia. In contrast infected female subjects were associated with each of the four regions and the biggest discrepancy was among subjects from northern and Saharan Africa, where prevalence among males was 0.0% (CL_95_ = 0.0–18.52), but 10.0% among females (CL_95_ = 6.20–15.43).

When all the species (protozoan and helminth) were combined, prevalence also varied significantly between the four regions (χ^2^_3_ = 8.76, *P* = 0.033) and the highest prevalence was again among those from northern and Saharan Africa (Table 2).

Since subjects from western Asia have been the particular focus of earlier studies [8, 9, 13], we present in Table 3 data on the prevalence of some of the key taxa broken down by nationality. Pakistanis were excluded because there were only five subjects in the study group.

**Table 3:** Prevalence % (CL_95_) of the most common taxa among subjects from western Asia

	*n*	Helminths combined	*A. lumbricoides*	Hookworms	Protozoa combined	*G. duodenalis*
Bangladesh	192	14.1 (8.51–22.22)	4.7 (1.86–10.69)	5.7 (2.53–11.92)	11.5 (6.43–19.12)	3.1 (0.94–8.39)
India	433	5.3 (3.07–8.95)	0.5 (0.11–2.42)	3.7 (1.85–6.94)	15.7 (11.53–20.90)	4.4 (2.35–7.80)
Nepal	373	6.2 (3.86–9.65)	1.1 (0.31–3.20]	4.3 (2.40–7.35)	13.7 (10.05–18.23)	4.0 (2.19–7.02)
Sri Lanka	286	6.6 (4.47–9.70)	1.0 (0.37–2.79]	5.6 (3.64–8.47)	8.0 (5.65–11.37)	2.1 (1.03–4.20)

Pakistan has been excluded because the study group only included 5 subjects from Pakistan

#### Prevalence of parasites by age class

**Fig. 1:**
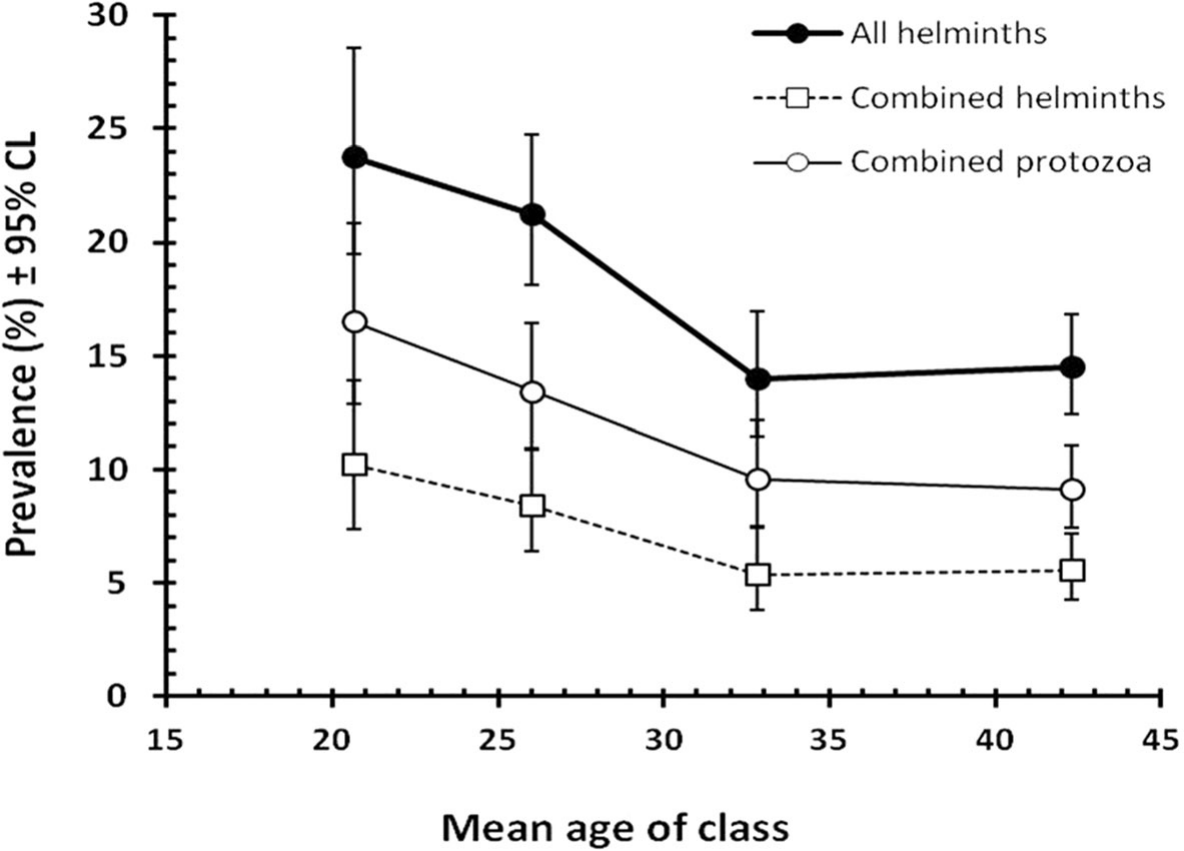
Change of prevalence of all the parasite species combined, combined helminths and combined protozoa with host age

Prevalence of all the parasite species combined varied significantly between the age classes (AGE CLASS x INFECTION, χ^2^_3_ = 19.8, *P* < 0.001), as did also that of combined helminths (χ^2^_3_ = 12.2, *P* = 0.007) and combined protozoa (χ^2^_3_ = 15.7, *P* = 0.001). In all three cases, prevalence was highest among the youngest age class, declining with age and stabilising among the two oldest age classes, as shown in Fig. 1. There was also a significant effect of age for hookworms (χ^2^_3_ = 11.2, *P* = 0.011) but not for *A. lumbricoides* (χ^2^_3_ = 2.6, *P* = 0.455) nor for *T. trichiura* (χ^2^_3_ = 3.86, *P* = 0.277).

Among the protozoa, *B. hominis* showed significant differences in prevalence between the age classes (when AGE CLASS was fitted alone with INFECTION, χ^2^_3_ = 8.47, *P* = 0.037), but this difference became negligible when other factors were taken into account. Similarly for *G. duodenalis*, models with AGE CLASS and INFECTION alone gave χ^2^_3_ = 9.8, *P* = 0.020, but models controlling for SEX and REGION, failed to identify AGE CLASS as a significant factor. Prevalence of *E. nana* and *E. coli* did not vary significantly between age classes.

#### Prevalence of parasites by host sex

No significant differences in prevalence between the sexes were detected for combined helminths, combined protozoa or all species combined, whether SEX was fitted alone with INFECTION or in multi-factorial models, although in all three cases prevalence was numerically slightly higher for male subjects. For combined parasites prevalence was 19.2% (CL_95_ = 16.92–21.50) in male subjects and 16.6% (CL_95_ = 14.60–18.56) in females and for combined helminths prevalence was 7.5% (CL_95_ = 5.98–9.26) in male subjects and 6.7% (CL_95_ = 5.36–8.19) in females. For combined protozoa, prevalence in male subjects was 12.7% (CL_95_ = 10.75–14.62) and 10.8% (CL_95_ = 9.15–12.46) in females.

For hookworms, a significant difference was detected when SEX was fitted with INFECTION alone and without other factors (χ^2^_1_ = 4.56, *P* = 0.033; prevalence in males = 4.4% [CL_95_ = 3.27–5.81]; prevalence in females = 2.8% [CL_95_ = 1.99–3.86]), but did not emerge as significant when models also controlled for REGION and AGE. No difference between the sexes was detected for prevalence of *A. lumbricoides* (males = 1.50% [CL_95_ = 0.87–2.40]; females = 2.0% [CL_95_ = 1.32–2.91]) or for *T. trichiura* (males = 0.97% [CL_95_ = 0.48–1.73]; females = 1.78% [CL_95_ = 1.14–2.64]), but interestingly in both cases prevalence was numerically slightly higher for female subjects.

There was no significant difference between the sexes in the prevalence of *B. hominis*, *E. nana* or *E. coli* when SEX was fitted alone with INFECTION. However, *G. duodenalis* was 4 times more prevalent among male compared with female subjects (males = 4.0% [CL_95_ = 2.89–5.31]; females = 1.0% [CL_95_ = 0.51–1.66]; SEX × INFECTION, χ^2^_1_ = 12.20, *P* < 0.001).

### Species richness

#### Number of helminth species

The mean number (± SEM) of helminth species per subject in the study group was 0.078 ± 0.006 with a range from 0 to a maximum of 3 species. Analysis of the number of helminth species harboured (GLM with a Poisson log-link) showed that only the effect of host age was significant (Wald χ^2^_3_ = 13.3, *P* = 0.004). The youngest members of the study group had the highest mean number of helminth species, followed by a reduction to age class 2 and a further reduction to the remaining older age classes. The highest regional mean was for the western Asians (Table 4), and the lowest for subjects from sub-Saharan Africa, and males had a slightly higher mean than females but these were not statistically significant effects.

**Table 4:** Mean number of helminth, protozoan and combined species (species richness) harboured per subject by region, age and sex

	Helminths	Protozoa	Combined
Region			
western Asia	**0.085** ± 0.009	0.166 ± 0.013	0.251 ± 0.016
eastern Asia	0.074 ± 0.009	0.105 ± 0.012	0.178 ± 0.016
northern & Saharan Africa	0.058 ± 0.020	**0.290** ± 0.054	**0.348** ± 0.056
sub-Saharan Africa	0.049 ± 0.020	0.163 ± 0.042	0.211 ± 0.049
Age (Age range)			
Age class 1 (16–22)	**0.116** ± 0.021	**0.201** ± 0.028	**0.317** ± 0.037
Age class 2 (23–29)	0.092 ± 0.011	0.169 ± 0.016	0.262 ± 0.019
Age class 3 (30–37)	0.061 ± 0.010	0.128 ± 0.015	0.188 ± 0.019
Age class 4 (38–58)	0.058 ± 0.011	0.121 ± 0.019	0.179 ± 0.021
Sex			
Male	**0.083** ± 0.009	**0.161** ± 0.014	**0.244** ± 0.017
Female	0.073 ± 0.008	0.140 ± 0.012	0.213 ± 0.015

The highest value in each data-subset is in bold

#### Number of protozoan species

The mean number (± SEM) of protozoan species per subject was 0.150 ± 0.009 with a range from 0 to a maximum of 4 species (harboured by just one individual). There was a significant difference between regions (Wald χ^2^_3_ = 13.6, *P* = 0.003), with the highest mean value for subjects from northern and Saharan Africa and the lowest for those from eastern Asia (Table 4). Values were also significantly higher for male subjects (Wald χ^2^_1_ = 4.27, *P* = 0.039) but there was also a significant interaction between SEX and REGION on the mean number of protozoan species/subject (Wald χ^2^_3_ = 11.0, *P* = 0.012). Whereas, among western Asian subjects (males = 0.18 ± 0.016; females = 0.13 ± 0.024) and those from sub-Saharan Africa (males = 0.17 ± 0.056; females = 0.16 ± 0.063) the mean number of species was approximately similar, in subjects from eastern Asia (males = 0.02 ± 0.012; females = 0.12 ± 0.014) and from northern and Saharan Africa (males = 0.17 ± 0.090; females = 0.31 ± 0.060), the mean values for the number of protozoan species harboured per subject were higher for female compared to male subjects.

#### Number of parasite species (helminths and protozoa combined)

The mean number (± SEM) of combined parasite species harboured per subject was 0.227 ± 0.011, with a range of 0 to 4 species. The regional effect was significant (Wald χ^2^_3_ = 13.7, *P* = 0.003) with the highest values derived from the northern and sub-Saharan Africans and the lowest from subjects from eastern Asia (Table 4). There was also a marked age effect (Wald χ^2^_3_ = 17.3, *P* = 0.001) with values declining from a maximum for age class 1 to the lowest value for age class 4. There was no significant difference in the number of parasite species harboured by male and female subjects.

### Abundance of infections

#### Abundance of parasites by region of origin of subjects

The only helminth species to show significant variation in abundance between regions was hookworm (*H*_3_ = 11.497, *P* = 0.009). Table 5 shows that the highest mean egg count was detected for western Asians and the lowest for subjects from sub-Saharan Africa. In contrast, among the protozoan parasites *B. hominis* (*H*_3_ = 9.295, *P* = 0.026), *E. nana* (*H*_3_ = 17.716, *P* = 0.001), *E. coli* (*H*_3_ = 30.25, *P* < 0.001) and *G. duodenalis* (*H*_3_ = 18.740, *P* < 0.001) all showed significant regional effects, which are summarized in Table 5. Combined helminths did not show a significant regional effect, but combined protozoa (*H*_3_ = 24.519, *P* < 0.001) and all parasites combined (*H*_3_ = 18.532, *P* < 0.001) did. The respective values are in Table 5.

**Table 5:** Abundance of helminth and protozoan parasites by region of origin of the subjects in the study

	Mean (Eggs/oocyst/cysts per gm of faeces) ± S.E.M. (Maximum Egg/Oocyst/cyst count)				
	western Asia	eastern Asia	northern & Saharan Africa	sub-Saharan Africa	Overall
Helminths					
*Ascaris lumbricoides*	3.88 ± 1.512 (1,433)	**5.28** ± 1.886 (1,373)	1.69 ± 1.596 (220)	4.50 ± 3.472 (400)	4.32 ± 1.075 (1,433)
Hookworms	**3.64** ± 0.851 (660)	1.11 ± 0.373 (200)	0.96 ± 0.587 (66)	0.64 ± 0.478 (53)	2.39 ± 0.466 (660)
*Trichuris trichiura*	**1.05** ± 0.442 (440)	1.04 ± 0.325 (166)	0.19 ± 0.188 (26)	0.21 ± 0.211 (26)	0.96 ± 0.260 (440)
*Strongyloides stercoralis*	0.08 ± 0.038 (33)	**0.15** ± 0.082 (66)	0 (0)	0 (0)	0.10 ± 0.037 (66)
*Hymenolepis nana*	0.97 ± 0.672 (833)	0.05 ± 0.043 (40)	**2.85** ± 2.848 (393)	0.76 ± 0.756 (93)	0.72 ± 0.385 (833)
All helminths combined	**9.93** ± 1.955 (1,499)	7.64 ± 1.964 (1,373)	8.04 ± 4.034 (393)	6.11 ± 3.574 (400)	8.77 ± 1.287 (1,499)
Protozoa					
*Blastocystis hominis*	**36.98** ± 6.279 (4,333)	17.99 ± 4.314 (2,533)	35.82 ± 12.099 (1,353)	22.64 ± 12.350 (1,033)	29.06 ± 3.753 (4,333)
*Endolimax nana*	32.53 ± 8.409 (7,333)	6.83 ± 2.106 (1,100)	36.89 ± 20.743 (1,986)	**102.15** ± 45.399 (4,293)	26.54 ± 5.113 (7,333)
*Entamoeba coli*	11.00 ± 3.169 (3,133)	4.42 ± 1.431 (720)	**16.22** ± 7.095 (820)	1.14 ± 0.873 (100)	8.32 ± 1.775 (3,133)
*Entamoeba histolytica*/*dispar*	**5.81** ± 2.267 (1,800)	1.37 ± 0.657 (420)	3.57 ± 2.799 (366)	0.65 ± 0.65 (80)	3.76 ± 1.212 (1,800)
*Giardia duodenalis*	**46.40** ± 9.714 (5,000)	8.04 ± 3.244 (1,800)	1.97 ± 1.928 (266)	0 (0)	27.19 ± 5.199 (5,000)
All protozoa combined	**134.83** ± 16.458 (9,786)	39.44 ± 6.510 (2,533)	119.82 ± 36.045 (3,392)^3^	127.01 ± 47.319 (4,293)	97.69 ± 9.436 (9,786)
Helminths and protozoa combined	**144.76** ± 16.537 (9,786)	47.08 ± 6.815 (2,533)	127.86 ± 36.079 (3,392)	133.11 ± 47.331 (4293)	106.47 ± 9.506 (9,786)

The highest regional prevalence for each species is in bold

Values are given for those taxa that showed an overall prevalence equal to or greater than 0.4%

#### Abundance of parasites by age of subjects

**Fig. 2:**
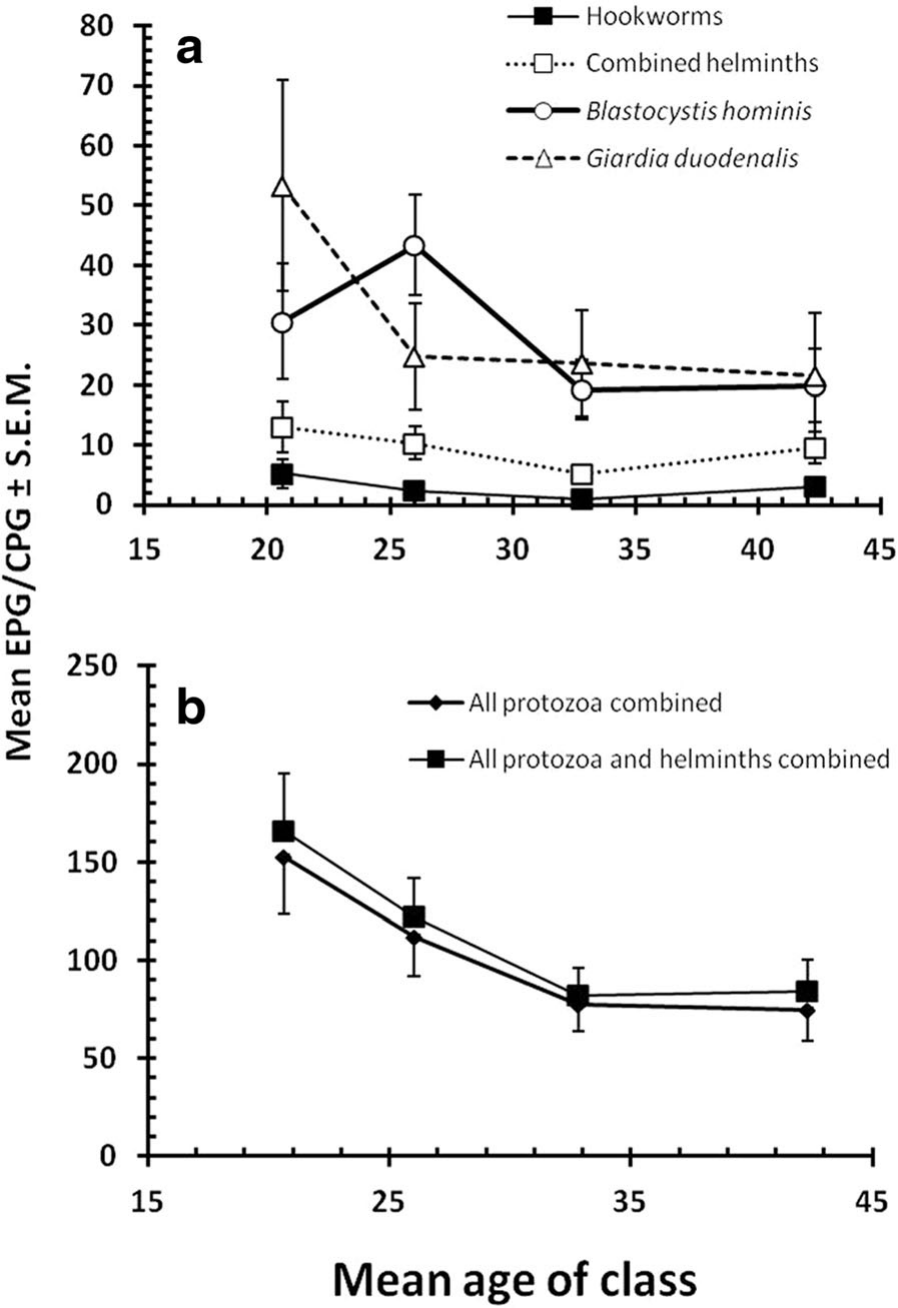
Change in abundance with host age of (**a**) hookworms, combined helminths, *B. hominis* and *G. duodenalis*, and **b** all protozoa combined and all protozoa and helminths combined

Among the helminth species, an age effect was seen only for hookworms (*H*_3_ = 13.3, *P* = 0.004) and for combined helminths (*H*_3_ = 12.353, *P* = 0.006). For hookworms, the mean egg count fell from age class 1 to age class 3 and then increased in the oldest age class (Fig. 2) and much the same pattern was found for combined helminths (Fig. 2). Protozoan species that showed a significant age effect were *B. hominis* (*H*_3_ = 8.889, *P* = 0.031) and *G. duodenalis* (*H*_3_ = 11.611, *P* = 0.009). There was also a significant age effect for combined protozoa (*H*_3_ = 16.450, *P* = 0.001) and for all parasites combined (*H*_3_ = 26.231, *P* < 0.001).

#### Abundance of parasites by sex of subjects

The only helminth species to show significant variation in egg counts between the sexes was hookworm (*z* = 2.142, *P* = 0.032), although the mean values were close (2.230 ± 0.436 EPG for males and 2.522 ± 0.775 for females). Among the protozoa only *G. duodenalis* showed a significant difference between the sexes (*z* = 4.950, *P* < 0.001). Cyst counts were much higher among male subjects (50.1 ± 10.79 CPG *vs* 8.0 ± 2.96 for females).

### Temporal effects

Recently Abu-Madi et al. [10] have shown that the prevalence of both helminth and protozoan infections has declined during the last decade among settled immigrants and residents in Qatar, and it was therefore, of interest to see whether any similar temporal effects were evident among the current dataset on recent arrivals.

#### Temporal effects on prevalence

**Fig. 3:**
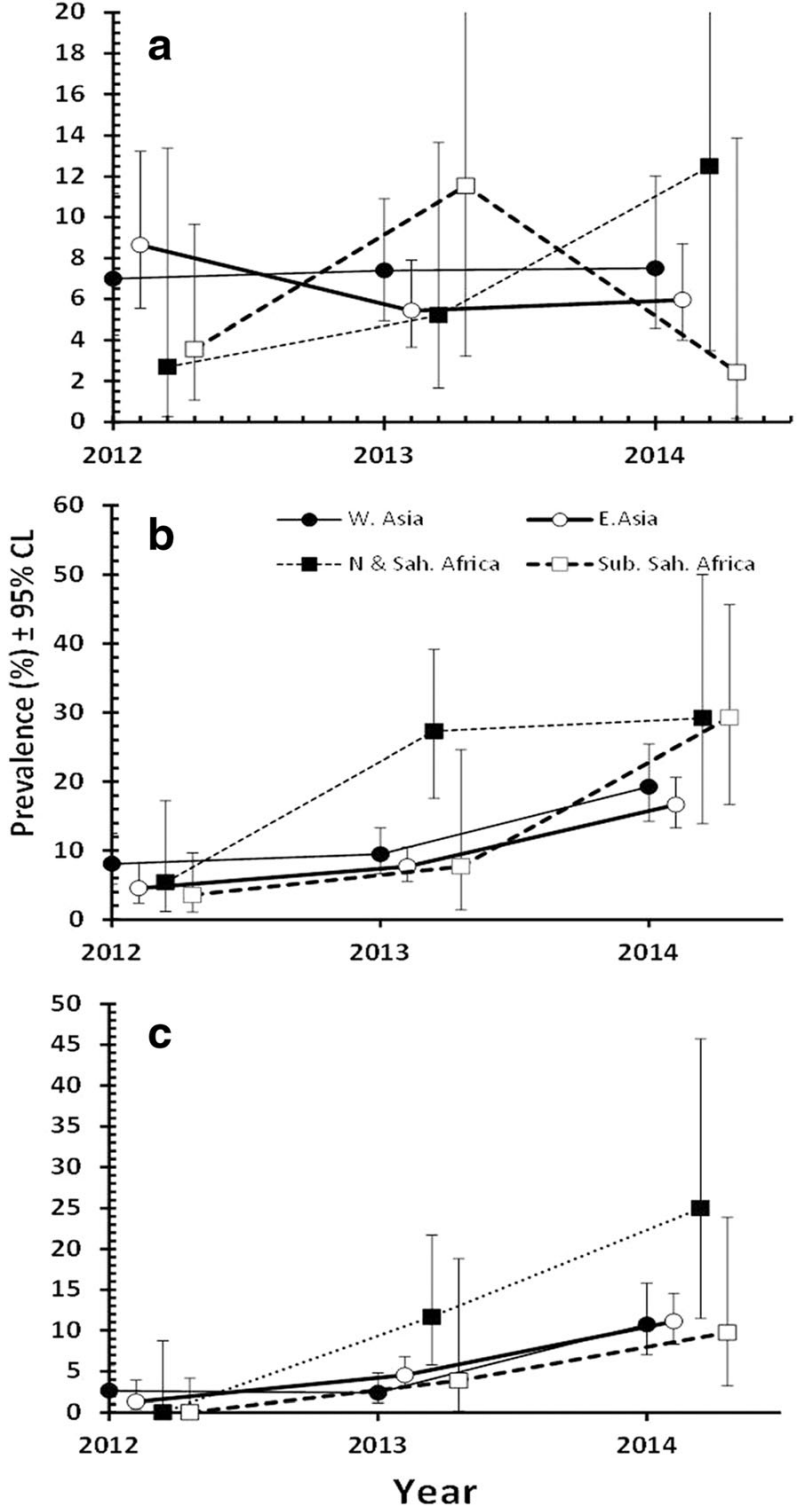
Temporal changes in the prevalence of (**a**) combined helminths, **b** combined protozoa and (**c**) *B. hominis*. Values along the X-axis are offset slightly to avoid overlap of error bars

Analysis of prevalence of combined helminths showed no evidence of any significant changes whether YEAR was fitted alone with INFECTION (for YEAR × INFECTION, χ^2^_2_ = 0.37, *P* = 0.830) or whether controlling also for REGION and AGE. The data in Fig. 3a show that prevalence for western Asians was very similar in each year of the study and that for eastern Asians showed a marginal decline between 2012 and 2013, but no further decline by 2014. The data for both African subsets showed more dynamic changes but these were based on relatively small sample sizes, and overall no significant change was found. We also examined independently the two species showing the highest overall prevalence (hookworms and *A. lumbricoides*). For hookworms, no temporal changes were evident whether INFECTION was fitted alone with YEAR or also with REGION and AGE. For *A. lumbricoides*, a significant temporal effect was found (YEAR × INFECTION, χ^2^_2_ = 12.1, *P* = 0.002, in a model that also took into account SEX and AGE) but prevalence did not follow a consistent trend across the 3 years, dipping in 2013 and then rising again in 2014 (for 2012 to 2014: 2.5% [CL_95_ = 1.60–3.62], 0.5% [CL_95_ = 0.22–1.18] and 2.0% [CL_95_ = 1.14–3.41], respectively).

In contrast, for combined protozoan infections a highly significant YEAR effect was detected (YEAR × INFECTION, χ^2^_2_ = 74.0, *P* < 0.001) in a model that also took into account the earlier detected significant effects of REGION and AGE and this followed an increasing trend over the 3 years. As Fig. 3b shows the prevalence of combined protozoan infections drifted marginally up between 2012 and 2013 for 3 regions but initially more sharply for subjects from northern and Saharan Africa. However, by 2014 all four regions showed markedly elevated prevalence compared to 2012.

A major contribution to the temporal changes of the combined protozoa came from *B. hominis* (Fig. 3c) which showed highly significant changes with increasing years of the study (YEAR × INFECTION, χ^2^_2_ = 81.5, *P* < 0.001, in a model that also took into account the significant effects of REGION and AGE). Although the temporal patterns of change in prevalence were not as clear-cut for other protozoan species, some also contributed to this increase in prevalence over the 3 years (e.g. prevalence of *G. duodenalis* in northern and Saharan Africans increased from 0.0% in 2012 to 4.2% in 2014; *E. nana* increased from 3.6% among sub-Saharan Africans in 2012 to 17.1% in 2014 and in the same regional group *E. coli* increased from 0.0% in 2012 to 4.9% in 2014). We also considered prevalence of *G. duodenalis* among west Asians, because of earlier reports that subjects from this region showed relatively high prevalence of this species [8, 11]. However, over the period 2012 to 2014, there was no significant increase in prevalence (YEAR x INFECTION, χ^2^_2_ = 3.8, *P* = 0.15), although numerically prevalence was higher in 2014 (4.9%, [CL_95_ = 2.59-9.73]) compared to in 2012 (2.8%, [CL_95_ = 1.28-5.99]) and 2013 (2.7%, [CL_95_ = 1.34-5.16]), and this would also have contributed to the overall increase in the prevalence of protozoan infection over this period.

#### Temporal effects on abundance

**Fig. 4:**
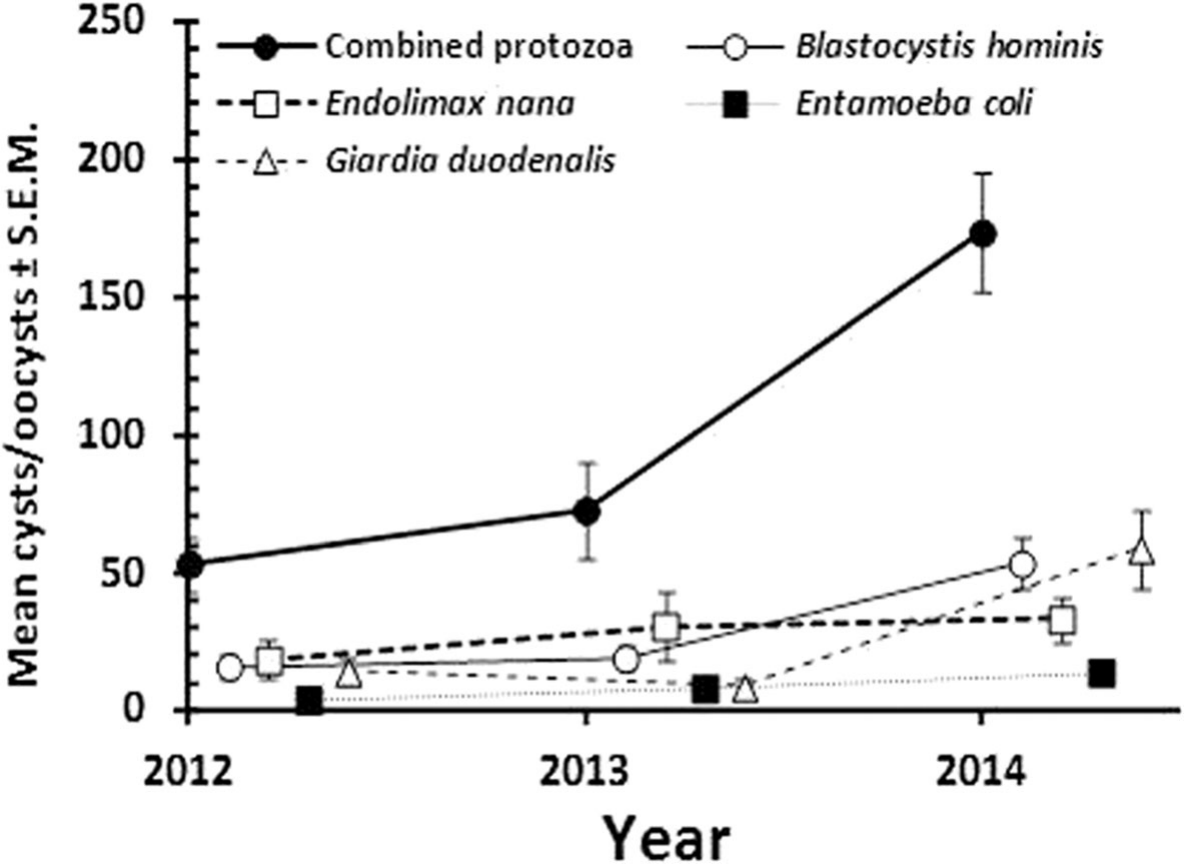
Temporal changes in the mean oocyst/cyst counts (in units of CPG) for combined protozoa, *B. hominis*, *E. nana*, *E. coli* and *G. duodenalis*. Values along the X-axis are offset slightly to avoid overlap of error bars

Quantitative analysis of faecal egg counts only revealed a significant effect of YEAR for *A. lumbricoides* (*H*_2_ = 9.584, *P* = 0.008). As with prevalence, the abundance of EPG showed a clear dip in 2013 relative to 2012 and 2014 (for 2012 to 2014, mean EPG = 5.20 ± 1.82, 1.40 ± 1.30 and 5.59 ± 2.14, respectively). Analysis of the oocyst/cyst counts also showed a significant effect of YEAR on combined protozoan counts (*H*_2_ = 75.9, *P* < 0.001), *B. hominis* (*H*_2_ = 78.3, *P* < 0.001), *E. nana* (*H*_2_ = 24.1, *P* < 0.001) and *E. coli* (*H*_2_ = 10.2, *P* = 0.006) but not for *G. duodenalis*. As shown in Fig. 4, mean CPG increased each year between 2012 and 2014 for the first four taxa but not in the case of *G. duodenalis*, which although showing increased CPG in 2014 relative to 2012, experienced also a dip in counts 2013.

#### Temporal effects on species richness

Helminth species richness did not vary significantly with year of study (for 2012 to 2014 the values were 0.077 ± 0.009, 0.079 ± 0.012 and 0.078 ± 0.011 species/subject, respectively). However for the protozoan species there was a significant increase in the mean number of species/subject across this period (Wald χ^2^_2_ = 30.2, *P* < 0.001). The values for 2012 to 2014 were 0.066 ± 0.008, 0.144 ± 0.018 and 0.259 ± 0.021, respectively.

## Discussion

In our earlier analyses of the prevalence of parasitic infections and their temporal trends among settled immigrants in Qatar [12], immigrants from western Asia were observed to harbor the highest prevalence of helminth infections whereas immigrants from most other regions lost their helminth burdens almost completely after acquiring residency permits. Given the huge numbers of immigrants from western Asian countries and the persistence of helminth infections among the Nepalese in particular, our findings were of some concern with respect to public health in Qatar and this was the intended focus of the current project. Therefore, over a period of 3 years (2012–2014) we monitored helminth and protozoan infections among randomly chosen newly arrived immigrants, each of which should have been tested for enteric parasitic infections, and treated accordingly if found to be infected, in the course of obtaining their PEC, prior to arrival in Qatar.

In contrast to the values for prevalence of helminths among long-term residents recorded in the period from 2005 to 2008, in subsequent years (2009–2011) a clear trend of declining prevalence was observed, but even so, subjects from western Asia, including Nepal, still showed the highest prevalence of helminth infections [12]. In the most recent analysis of infections among long-term residents (including 2012–2014), prevalence of helminth infections was found to have continued to fall even further and the distinction between residents from western Asia and other regions had become less obvious [10]. Thus, until 2014 falling prevalence of intestinal parasitic infections was seen as evidence of the success of Qatar’s policies. Indeed, it is now mandatory for all applicants for entry to the country for work, to undergo health checks in their country of origin and to obtain a PEC.

The overall prevalence of parasites (both helminths and protozoa combined) in the current study based on freshly arrived immigrants was 17.8% which is the lowest value observed so far for immigrants to Doha. Among recently arrived immigrants a prevalence of 33.9% was observed in the period 2005–2006 [13] and in 2009 a prevalence of 26.5% [8], in both cases for combined helminth and protozoan infections. The current value is also lower than prevalence reported elsewhere, as for example among new immigrants to Australia (39.03%; [17]), and most likely again reflects Qatar’s success in curbing infections carried into the country by legislation requiring mandatory health checks before arrival. However lower prevalences have also been observed elsewhere as for example among immigrants in Spain with a prevalence of just 12.7% [7].

The highest prevalence of helminths in the current study was observed among the western Asia group (7.3%) but only just higher than that among the eastern Asians (7.2%), supporting the current picture of not only falling prevalence but also less distinction between the regions. As explained earlier, in some respects the higher prevalence values for Asian immigrants are not surprising given that Nepalese, Bangladeshi, Indian and Sri Lankan immigrants constitute the largest immigrant work force in Qatar (Medical Commission database) and given the poverty associated with these countries and the documented prevalence of helminth infections among rural communities in western Asia [18]. The value for prevalence for combined helminth infections was largely driven by hookworms among western Asians which showed a declining trend throughout the years of close monitoring, from 11.25% in 2005 and 6.0% in 2009 to 4.7% during 2012–2014 among newly arrived immigrants. The overall prevalence of helminths was lower than those reported in Spain with 15.4% [6] and in New Mexico with 19.3% [19]. In Australia, a study reported significantly more frequent prevalence of intestinal helminths among short-term immigrants than in medium- or long-term immigrants (25%, 14.8% and 4.6%, respectively) [20]. This is consistent with earlier studies comparing prevalence among newly arrived immigrants with that among residents [8] and with the current work (prevalence of helminths among newly arrived immigrants in the current work was 7.0% and among residents was reported to be 0.93% [10]).

A quite different pattern was observed for protozoan infections. The most prevalent protozoan parasites were *B. hominis* among north and Saharan African subjects and *G. duodenalis* among western Asia subjects. In contrast to the generally falling trends in prevalence of intestinal protozoan infections among long-term residents [11], the current data revealed an increasing trend in the short-term over the period 2012–2014 among new arrivals. Comparing the current findings with the previous analysis in 2009 [8], an increase is apparent: from 3.4 to 10.9% for *B. hominis* among northern Africans and 1.4 to 3.6% for *G. duodenalis* among western Asians. Whether this upward trend in prevalence of *B. hominis* is real and likely to be sustained or just a temporary phenomenon will only become apparent in time. However, it is pertinent that among settled immigrants in Qatar, the prevalence of *B. hominis* (3.5% across the period from 2005 to 2014) varied significantly between years but did not show any significant long-term directional change over time. African subjects seem to be more prone to *B. hominis* infection, in agreement with a report from Australia in which *B. hominis* was identified more frequently among African immigrants than Asian [17]. The prevalence of this intestinal parasite among the north and Saharan African group in the current study is similar to that found in previous studies on migrant African populations elsewhere in the world [20].

*Giardia duodenalis* was more commonly encountered among western Asians as observed in earlier work [8, 11]. Analysis of prevalence over a whole decade showed that this species declined with host age [11] but a resurgence of *Giardia* infection among Asian young male subjects was observed suggesting continued exposure throughout life. The affected individuals are most likely to be the unskilled construction workers aggregating in overcrowded labour camps where conditions may be cramped and sanitary facilities limited and of a poor standard. When comparing prevalence in the current report with an earlier analysis in 2009, an increase is apparent from 1.4% [8] to 3.6% (Table 2 in current report) among western Asians, however, there was no significant increase over the 3 year period from 2012 to 2014 among recent west Asian immigrants in the current data-set. Higher prevalence has been reported elsewhere as for example among new immigrants in Spain with 5.4% [6]. The earlier data suggest that some transmission may occur in Qatar, as is evident by its presence also in Qatari subjects who are not immigrants [11], and this is a worrying concept for local public health in the country.

## Conclusions

The increase of protozoan infections over recent years, during which the current study was conducted, raises some concerns. In contrast to the helminths which appear to be increasingly well controlled among immigrants prior to their arrival in Qatar, as adjudged by the current data, protozoan infections among new arrivals appear to be increasing, at least in the short-term. This is of particular relevance among immigrants who then go on to work in the food handling industries and as housemaids. It suggests that the screening protocols for applicants for visas need to be revised and even reinforced by more careful attention to the intestinal protozoan infections that potential immigrants may harbor. Therefore, mandatory checks for intestinal protozoan infections and subsequent treatment with anti-protozoal agents prior to arrival may be a desirable course of action for the future, and we strongly recommend that Qatar’s health authorities implement such measures in the near future.

## References

[1] Mezeid N, Shaldoum F, Al-Hindi AI, Mohamed FS, Darwish ZE (2014). Prevalence of intestinal parasites among the population of the Gaza Strip, Palestine. Ann Parasitol.

[2] Tsiodras S (2016). Irregular migrants: a critical care or a public health emergency. Intensive Care Med.

[3] UNFPA, 2013. http://www.unfpa.org/sites/default/files/pub-pdf/CEB%20Pub.%20International%20Migration%20and%20Development.pdf.

[4] IOM, 2015. https://www.iom.int/sites/default/files/our_work/ICP/MPR/WMR-2015-Background-Paper-CSchultz.pdf.

[5] CDC, 2013. http://www.cdc.gov/immigrantrefugeehealth/pdf/intestinal-parasites-overseas.pdf.

[6] Monge-Maillo B, López-Vélez R, Norman FF, Ferrere-González F, Martínez-Pérez A, Pérez-Molina JA (2015). Screening of imported infectious diseases among asymptomatic Sub-Saharan African and Latin American immigrants: A public health challenge. Am J Trop Med Hyg.

[7] Lopez-Velez R, Huerga H, Turrientes MC (2003). Infectious diseases in immigrants from the perspective of a tropical medicine referral unit. Am J Trop Med Hyg.

[8] Abu-Madi MA, Behnke JM, Ismail A, Al-Olaqi N, Al-Zaher K, El-Ibrahim R (2011). Comparison of intestinal parasitic infections in newly arrived and resident workers in Qatar. Parasit Vectors.

[9] Abu-Madi MA, Behnke JM, Doiphode SH (2010). Changing trends in intestinal parasitic infections among long-term-residents and settled immigrants in Qatar. Parasit Vectors.

[10] Abu-Madi MA, Behnke JM, Boughattas S, Al-Thani A, Doiphode SH, Deshmukh A (2016). Helminth infections among long-term residents and settled immigrants in Qatar in the decade from 2005 to 2014: temporal trends and varying prevalence among subjects from different regional origins. Parasit Vectors.

[11] Abu-Madi MA, Behnke JM, Boughattas S, Al-Thani A, Doiphode SH (2016). A decade of intestinal protozoan epidemiology among settled immigrants in Qatar. BMC Infect Dis.

[12] Abu-Madi MA, Behnke JM, Doiphode SH (2013). Intestinal parasitic infections among long-term-residents and settled immigrants in Qatar in the period 2005 to 2011. AJTMH.

[13] Abu-Madi MA, Behnke JM, Ismail A (2008). Patterns of infection with intestinal parasites in Qatar among food handlers and housemaids from different geographical regions or origin. Acta Trop.

[14] Bush AO, Lafferty KD, Lotz JM, Shostak AW (1997). Parasitology meets ecology on its own terms: Margolis et al., revisited. J Parasitol.

[15] Margolis L, Esch GW, Holmes JC, Kuris AM, Schad GA (1982). The use of ecological terms in parasitology (report of an ad hoc committee of The American Society of Parasitologists). J Parasitol.

[16] Rohlf FJ, Sokal RR (1995). Statistical Tables.

[17] Caruana SR, Kelly HA, Ngeow JYY, Ryan NJ, Bennett CM, Chea L, Nuon S, Bak N, Skull SA, Biggs BA (2006). Undiagnosed and potentially lethal parasite infections among immigrants and refugees in Australia. J Travel Med.

[18] Khanal LK, Choudhury DR, Rai SK, Sapkota J, Barakoti A, Amatya R (2011). Prevalence of intestinal worm infestations among school children in Kathmandu. Nepal Nepal Med Coll J.

[19] Skeels MR, Nims LJ, Mann JM (1982). Intestinal parasitosis among Southeast Asian immigrants in New Mexico. AJPH.

[20] Rice J, Skull S, Pearce C, Mulholland N, Davie G, Carapetis J (2003). Screening for intestinal parasites in recently arrived children from East Africa. J Paediatr Child Health.

